# Rapid and specific processing of person-related information in human anterior temporal lobe

**DOI:** 10.1038/s42003-018-0250-0

**Published:** 2019-01-04

**Authors:** Artem Platonov, Pietro Avanzini, Veronica Pelliccia, Giorgio LoRusso, Ivana Sartori, Guy A. Orban

**Affiliations:** 1 Department of Medicine and Surgery, University of Parma, via Volturno 39E, 43125 Parma, Italy; 2 Institute of Neuroscience, CNR, via Volturno 39E, 43125 Parma, Italy; 3 Claudio Munari Center for Epilepsy Surgery, Niguarda Hospital, Ospedale Ca’Granda Niguarda, Piazza dell’Ospedale Maggiore, 3, 20162 Milan, Italy

## Abstract

The anterior temporal lobe (ATL), located at the tip of the human temporal lobes, has been heavily implicated in semantic processing by neuropsychological and functional imaging studies. These techniques have revealed a hemispheric specialization of ATL, but little about the time scale on which it operates. Here we show that ATL is specifically activated in intracerebral recordings when subjects discriminate the gender of an actor presented in a static frame followed by a video. ATL recording sites respond briefly (100 ms duration) to the visual static presentation of an actor in a task-, but not in a stimulus-duration-dependent way. Their response latencies correlate with subjects’ reaction times, as do their activity levels, but oppositely in the two hemispheres operating in a push-pull fashion. Comparison of ATL time courses with those of more posterior, less specific regions emphasizes the role of inhibitory operations sculpting the fast ATL responses underlying semantic processing.

## Abstract (web summary)

Platonov et al. performed intracerebral recordings from twenty-four drug-resistant epilepsy patients during a discrimination task aimed at discerning actions or gender-related information. They showed that the anterior temporal lobe was specifically activated for 100 ms when patients discriminated the gender of the actor on the static frame presented before the videos.

## Introduction

Humans rapidly categorize conspecifics as female or male primarily using face information but also hand and body information^1,2^. Which neuronal processes underlie this task? Functional imaging studies have implicated various face processing regions^3^, most prominently the occipital face area (OFA) and fusiform face area (FFA)^4^. Electrophysiological studies using scalp recordings (Electro-Encephalography, EEG), however, have identified evoked-response potential (ERP) components, distinct from the N170 associated with FFA, and that have been assigned to more anterior temporal regions^2^. Given the technical difficulty of imaging BOLD in the rostral part of the temporal lobe^5^, it is possible that these more rostral region have escaped most of the imaging investigations so far. Recently, however, a rostral face region, the anterior temporal face patch (ATFP), has been identified^6,7^. This region is involved in face recognition^8^ and has been implicated in congenital prosopagnosia^9^, yet its role in gender discrimination has not been assessed. Stereo-EEG (sEEG), i.e., intracerebral recordings performed in drug-resistant epilepsy patients for diagnostic purposes, has millisecond temporal resolution as does its scalp counterpart, but has the advantage of directly localizing electrical signals reflecting neuronal activity in gray matter. We have further perfected its analysis^10^ by integrating data from large cohorts of patients onto a common template, allowing us to survey most of the cerebral hemispheres. This technique thus seemed ideal for investigating the neural substrate of gender categorization. We particularly wanted to study the involvement and functional properties of the anterior temporal lobe (ATL), a key region for the semantic processing^11^ of many different categories^12^ (but see ref. ^13)^, including human-social categories and person identity^14,15^. Although left and right ATL are thought to function as a single hub^11^, neuropsychological studies have abundantly documented the specialization of right and left ATL in visual/face versus verbal/voice processing, respectively^16^. We used a task-based attentional modulation paradigm^17,18^ in which subjects performed two different tasks on the same visual stimulus sequence. Subjects viewed a video portraying one of the two actions performed by a male or female actor, preceded by the static presentation of the first video frame for a variable duration, and discriminated either the action or the actor. In gender categorization studies many different exemplars of female and male subjects are shown, while we presented only a single male and female actor, to match the number of alternatives in the two tasks. It is conceivable that the patients distinguished between the two individuals, using a visual mechanism such as that described for face identity in monkey anterior medial (AM) patch^19^, rather than between genders. To remain cautious, we therefore refer to the task of interest as an actor discrimination task, which can be solved by either a semantic mechanism representing the gender categories or a visual mechanism representing the individuals. Our results show that performing the actor discrimination task does in fact activate the ATL, suggesting that subjects access the semantic categories of male and female in this simple task. Hence we were able to describe the unexpectedly short time scale on which the ATL operates, as well as its task specificity, two properties rooted in strong inhibitory mechanisms active in ATL

## Results

### Localization of selective leads

**Fig. 1:**
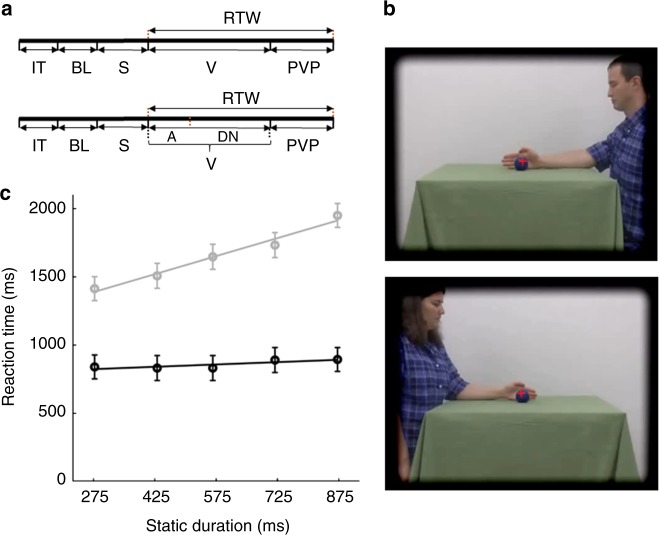
Task and behavior. **a** Trial structure; **b** original and inverted first frames (identical for the two actions) with fixation cross; **c** RTs of 24 patients for actor (black) and action (gray) discrimination plotted as a function of stimulus duration. Error bars indicate SD; two-way ANOVA (task × duration): main effect task F_1, 2752_ = 4642, *p* < 0.01, duration F_4, 2752_ = 84.5, *p* < 0.01, interaction F_4, 2752_ = 51.5, *p* < 0.01; the slope for actor discrimination was very shallow (0.14). IT: inter-trial interval (1 s), BL: baseline (1 s), S: static frame presentation (variable 575 ms +/−300 ms), V: video presentation (1.166 ms), PVP: post video period (2 s), RTW: reaction time window, A: action (200–250 ms + /−100 ms), DN: dynamic noise

**Fig. 2:**
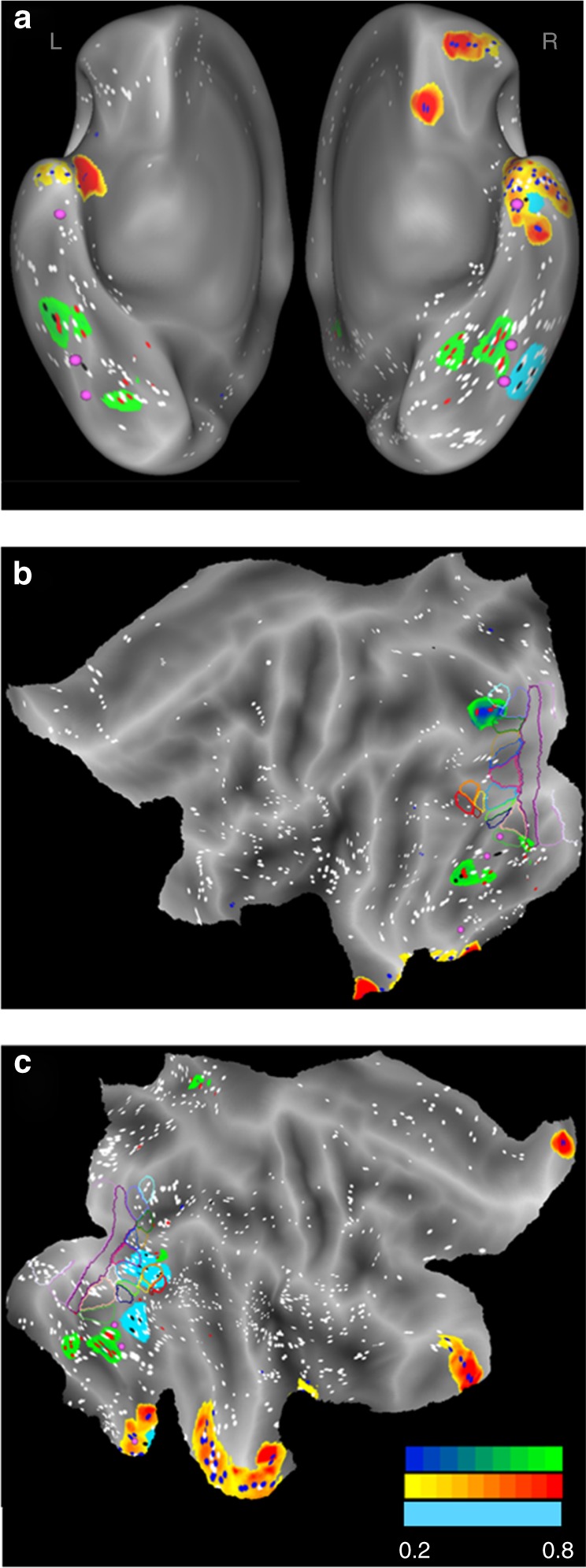
Localization of doubly-, static- and task-specific leads. Doubly-specific regions in orange (blue dots), static-specific regions in green (red dots), and task-specific regions in cyan (black dots) are shown on ventral views of the left and right hemispheres (**a**) and flat maps of left (**b**) and right hemisphere (**c**). White dots: other responsive leads. Filled pink circles: anterior and posterior FFA patches, and anterior temporal face patch. Colored outlines: retinotopic regions according to Abdollahi et al.^21^. Color code indicates relative responsiveness (20–80%) of the three types of specific leads

We obtained intracerebral recordings from 24 epilepsy patients (14 right, 7 left, 3 bilateral implantations, Supplementary Tables 1, 2, 3) discriminating either actions or actors (both 97% correct) in stimulus sequences of 1.166 s videos (Fig. 1a) preceded by the static presentation of the first video frame. The videos showed two actors (one male, one female, Fig. 1b) performing two different actions (dragging, grasping), but the first static frame was identical for the two actions. The static presentation lasted from 275 to 875 ms, to disentangle responses to static and video presentations. In a 2 × 2 design, subjects also performed the tasks in ‘short’ trials, identical to the standard ones, except that the video duration was reduced to 100–350 ms (adapted to individual thresholds determined before recording, see methods), with the remainder of the video replaced by dynamic noise (Fig. 1a). The reaction times (RTs) for actor discrimination were shorter (857 ms) and less dependent on static stimulus duration than those of action discrimination (Fig. 1c). This finding, which also held for short trials (Supplementary Fig. 1), indicates that subjects based their decision about the actor on the static presentations. Hence we searched amongst the many overall-responsive leads (*n* = 1023 in right (R) and *n* = 491 in left (L) hemispheres) (Supplementary Figs 2, 3), for those responding only during the static phase and specifically involved in the actor task. The great majority of these doubly-specific leads (46/62) were located in ATL (Supplementary Table 3), mostly right ATL (*n* = 36, 8 patients) but also left (*n* = 10, 4 patients), as well as right (*n* = 9) and left (*n* = 1) orbito-frontal cortex (OFC). This distribution contrasted sharply with that of leads showing only one such specificity (Supplementary Fig. 4), which were located more posteriorly in occipital, temporal and parietal cortex (Fig. 2). Leads specific only for the static epoch (static-specific, 29 in R and 14 in L hemisphere) were located in fusiform cortex (FG, 9R and 6L, 8 patients), at the level of the FFA^20^ in the right hemisphere, but more rostrally in the left, in addition to locations in the collateral sulcus, lateral occipital cortex, and posterior intraparietal sulcus (IPS). Leads with only task dependent responses (task-specific, 13R and 3L) were mostly located laterally in the occipito-temporal cortex (OTC) of the right hemisphere (*n* = 10, 4 patients), more precisely at the level of retinotopic middle temporal (MT)/lateral-occipital 2 (LO2) areas^21^ and in posterior occipito-temporal sulcus (pOTS) in front of the putative human posterior infero-temporal (phPIT) pair. Most of the remaining task-specific leads were located in more ventral temporal regions. Both task and static specificities were greater in the doubly-specific leads than in their single counterparts (Supplementary Figs. 4, 5). These results show the considerable functional specificity of ATL leads.

### Time course of activation in specific leads

**Fig. 3:**
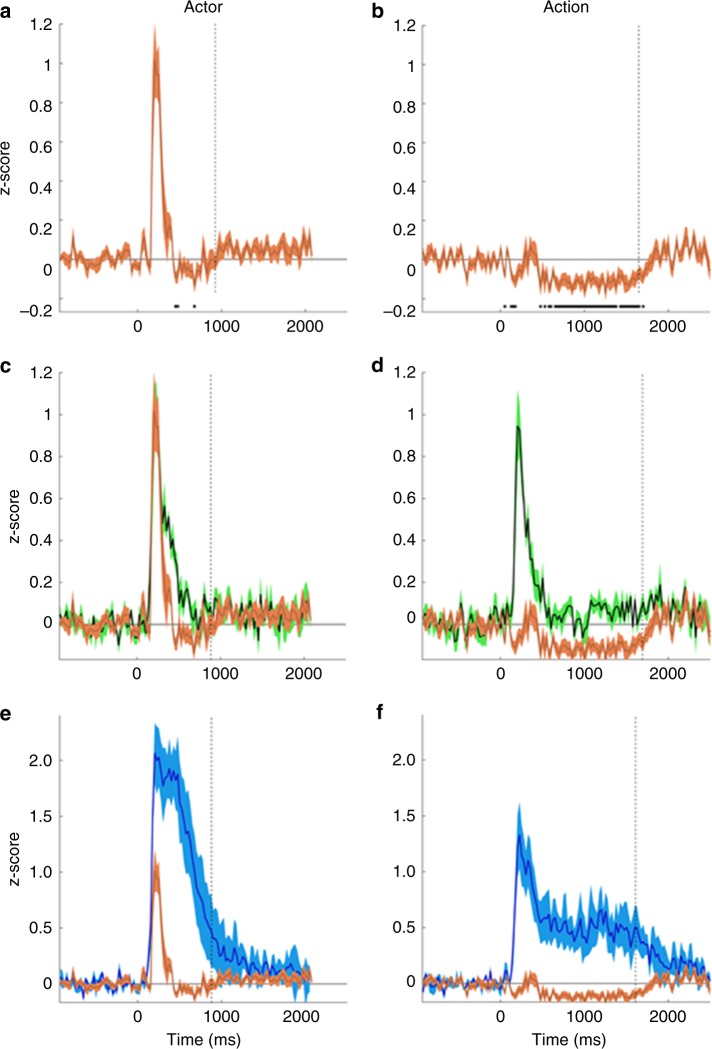
Average (across subjects) ATL time courses. **a**, **b** Average time courses of doubly-specific ATL leads (*n* = 12, orange) in actor (**a**) and action (**b**) discrimination trials; **c**–**f** Average time courses of doubly-specific ATL (*n* = 12, orange) and static-specific FG (*n* = 8, green) leads compared (**c**, **d**), and of doubly-specific ATL (*n* = 12, orange) and task-specific OTC (*n* = 4, blue) leads compared (**e**, **f**) in actor (**c**, **e**) and action (**d**, **f**) discrimination trials. Time zero is onset of the static frame. Vertical lines indicate mean RTs corresponding to the end of stimulation, hatching SEs, and horizontal black line segments in **a**, **b**, significant inhibition in the 1000 ms (**a**) and 1750 ms (**b**) after static onset (one-tailed *t-*test from zero, Bonferroni corrected for 40 and 70 comparisons)

ATL leads also had a very distinct temporal behavior. They responded very briefly in the actor discrimination task but not at all during action discrimination, exhibiting complete task-dependency. The average time course indicates that ATL responses in actor discrimination are terminated by suppressive effects (Fig. 3a), which likely explain the short responses in ATL. These suppressive effects were even more prominent during action discrimination, producing an absence of ATL responses. In this task a strong suppression, starting 100 ms after static onset, maintained the incoming activation below baseline, and lasted until the patient responded, which ended stimulus presentation (Fig. 3b). Thus while the ATL was completely silent during action discrimination, the strong suppression suggests that visual signals still reach this structure during this task. As a consequence of these inhibitory inputs, responses during action discrimination, present in more posterior FG and OTC leads, disappeared in ATL (Fig. 3d, f).

ATL responses during actor discrimination started shortly after those in FG (Fig. 3c), which is connected to ATL by a white-matter tract^22^. The latencies of these ATL activations were quite consistent within subjects (mean range 6 ms) but varied widely between subjects (range 67 ms). Hence we calculated the individual subjects’ latencies (*n* = 12) and report their distribution: mean 179 ms, SD = 21 ms. The FG latencies (*n* = 8) were shorter: mean 169 ms, SD 19 ms, but the difference from ATL was not significant: two-tailed *t*-test, *t*_18_ = 0.73, *p* > 0.45. The OTC latencies were much shorter (Fig. 3e, mean 135 ms, SD = 18 ms), and differed significantly from those of the ATL leads (two-tailed *t*-test, *t*_14_ = 3.81, *p* < 0.005). Importantly, ATL activations during actor discrimination finished earlier than FG activations (Fig. 3c) and much earlier than the OTC responses (Fig. 3e). Although ATL response durations were more variable than ATL latencies within subjects (mean range 32 ms), these within-subject variations were again smaller than inter-subject differences (132 ms range). Hence we calculated the distribution of the individual durations, the mean being 104 ms (SD 38 ms) in ATL compared to 148 ms (SD 50 ms) in FG, a difference which reached significance: two-tailed *t*-test, *t*_18_ = 2.38, *p* < 0.05. Yet both ATL and FG activations during actor discrimination were clearly shorter than the average static presentation (575 ms). In contrast, the OTC leads remained active until the subject responded, when the stimulus was switched off, and lasted more than 900 ms (Fig. 3e).

**Fig. 4:**
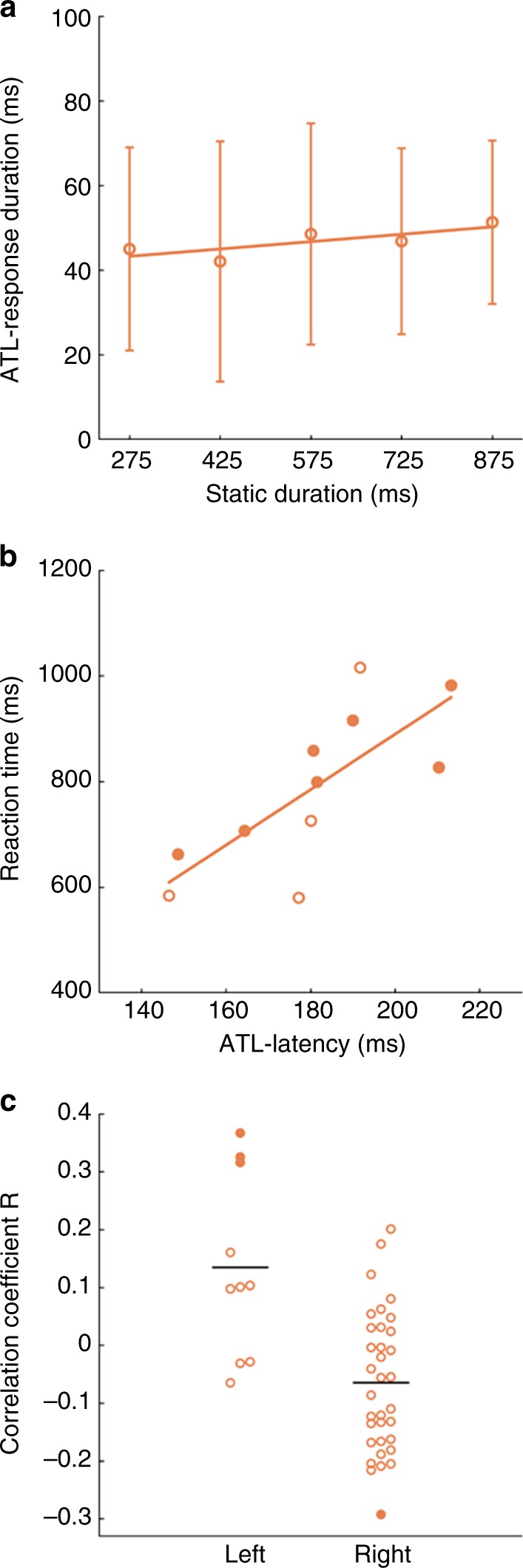
Properties of ATL responses. **a** Relationship between mean individual ATL response durations and stimulus duration (error bars = SD across subjects); **b** correlation of average ATL latencies and RTs in 11 subjects; open circles: left ATL; filled circles: right ATL; **c** dot plots of correlation coefficients between RTs and gamma power of left and right ATL leads: open and filled circles non-significant and significant leads, respectively. Notice that in **a** response durations are shorter when calculated for the five different static-frame durations independently than for all static durations pooled (see text). In **b**, **c** data of only 11 patients were analyzed (see methods), hence the numbers of leads in R and L hemisphere (**c**) are 34 and 10, respectively

Not only were the ATL responses very brief, much shorter than documented earlier in a semantic task^23^, but the response duration did not depend on the stimulus duration: for all static frame presentation times, the mean ATL response duration was close to 50 ms (Fig. 4a). ATL latencies and durations were similar in short trials (Supplementary Fig. 6). OFC leads showed a specificity similar to that of ATL leads (Supplementary Fig. 7) with responses that were as short as, or shorter than those in ATL.

### Relation of ATL leads with behavior

We also investigated the relationship between ATL responses and behavior. The latency of the ATL responses in individual patients (*n* = 11) correlated with their RTs (Pearson correlation, *r* = 0.75, *p* < 0.01, Fig. 4b). The correlation was positive, indicating that patients in whom ATL doubly-specific leads responded early also had shorter RTs. Such correlation was weak for the FG leads (Pearson correlation, *r* = 0.43, ns), and had the opposite sign (Pearson correlation, *r* = −0.49, ns) in OTC, although the number of patients was small, particularly for the OTC. Interestingly, similar correlations were observed in the short trials (Supplementary Fig. 8), for which the correlation in ATL was even more significant (Pearson correlation, *r* = 0.83, *p* < 0.01). Furthermore, to show the relationship of single-lead activity to behavior, inspired by choice probabilities^24^, we exploited the inter-trial variation in activity. The correlation between the activity levels of individual leads and RTs across trials reached significance in 4/46 ATL leads (Fig. 4c). While the degree of correlation is within the range reported earlier for frontal intracranial recording^25^, and the number of significant leads is small, it is important to note that the sign of the correlation differed significantly between hemispheres. The mean correlation equaled −0.06 (SD 0.12) in right ATL and 0.13 (SD 0.16) in left ATL (two-tailed *t*-test, *t*_42_ = 4.27, *p* < 0.01). Thus, increased activity in right and left ATL had opposite effects on RTs, with higher activity in the right ATL decreasing the RT, and increasing the RT in left ATL. This R–L difference remained significant when original (two-tailed *t*-test, *t*_42_ = 3.8, *p* < 0.01) and inverted frames (two-tailed *t*-test, *t*_42_ = 3.71, *p* < 0.01) were considered separately.

## Discussion

Our results indicate that in the right hemisphere, where most if not all regions involved in face processing^6^ have been explored, the ATL presents a unique functional profile being both task- and static-specific (see Supplementary notes, Supplementary Figs. 9 and 10), a feature shared only with the OFC. The same observation applies to the left ATL, although the left hemisphere has been less well explored in the present study. These properties reflect the behavior of the patients, who based their decisions in the actor discrimination task on the static epoch. Furthermore, the latency of the ATL activation correlated with the reaction times of the patients. Our results also provide clues about the visual information upon which the ATL operates during actor discrimination. The ATL shares with fusiform cortex the property of responding only to static stimuli. This is likely a consequence of the deleterious effect of motion on visual acuity^26,27^ and perceived position^28^. These psychophysical results suggest that regions providing detailed analysis of the various shape characteristics of the face, as described by Chang and Tsao^19^, may operate only on static stimuli, as we observed in fusiform and ATL regions. The static-specific regions documented in the present study are more restricted and located more anterior in the temporal lobe than sEEG responses to passive face presentations^29^. The stimuli shown here contain a second source of gender information, the hands which are the effectors of the action. We propose that this moving body-part-shape information is processed by the occipito-temporal task-specific regions documented in the present study. Indeed, the MT cluster overlaps with the extrastriate body area (EBA)^30^, and the representation of the upper limb is quite extensive in these regions^31^, providing ample opportunity for integrating motion and body parts. Furthermore, the pOTS region corresponds to the region driven by the configuration aspect of biological motion^32^. The anatomy of the ventral stream in the macaque^33,34^ suggests that this moving hand information converges with static face information upon ATL. Such view of convergent visual inputs to ATL is supported by the observation of Chatterjee and Nakayama^35^ that gender discrimination is intact in prosopagnosia, because a third source of information, not present in our stimuli, provided by faces in motion (e.g., in emotional expressions or vocal communication), reaches ATL by a segregated route in the Superior Temporal Sulcus^36–38^.

At the onset of the study we asked which cognitive strategy the patients were using to solve the actor discrimination task. The posterior part of the right ATL activation overlaps with the ATFP^8^ which has been suggested^33^ to be the homolog of the AM patch in the monkey, where neurons encode the visual identity of the face^19^. However, it is unclear whether patients base their decisions solely on the perceptual face-identity information, processed at that level. Indeed, many of the activated ATL leads are located more rostrally in both hemispheres, and hence might correspond to regions representing the concepts of male and female humans. These semantic regions, at the apex of the ventral stream^34^ may receive, as suggested above, convergent information about static faces (from the leads near ATFP) and moving hands (from the occipito-temporal task-specific region), and could have been triggered by the instructions to the patients, explicitly mentioning gender. This view is consistent with the finding that suppression of the ATL by transcranial magnetic stimulation reduces implicit gender-stereotypes for which gender categorization is a prerequisite^39^. These earlier studies indicate that the standard gender categorization task, in which multiple exemplars are shown, involves the ATL where social categories are represented. The present study suggests that this was also the case for the actor discrimination task, even if only a single exemplar of each category was presented.

The ATL processing of the static video frame is rapid, with latencies below 200 ms, in agreement with the intracranial recordings^40,41^, and also the EEG data^2,42^. These latencies correspond to the time at which information provided by eyes and mouth, the primary face-gender cues, is maximal in behavioral studies^43^. Even more remarkable is the short duration (100 ms) of the ATL activation. This cannot be measured in ERPs which analyze the latencies of time course extrema, and whose time course is very different from that of gamma power (see Supplementary Information in ref. ^10^). This finding indicates that the ATL can process several visual items in a single fixation, which may account for the speed of visual categorization^44^, and address the computational load of this structure that is involved in so many aspects of semantic processing^11–13^. Our study opens the possibility of investigating the time course of multiple semantic domains and levels of categorizations^45^.

The visual processing in ATL differs considerably from that in the more posterior regions, both the occipito-temporal task-specific region and the static-specific fusiform region. ATL processing is more abstract, being completely dependent on task and not at all on stimulus-duration, and relates to behavior. Particularly interesting is the difference from the fusiform region, which likely connects directly with ATL, as suggested by the anatomy^22^ and the 10 ms latency difference, consistent with a direct connection^46^. Such large transformations between one area and the next have been documented at lower levels of the visual system, e.g., between V1 and MT^47^ or between MT and the medial superior temporal (MST) area^48^, and have been shown to rely on nonlinear processing and the pattern of convergent afferents^49^. Here in contrast, the transformations rely heavily on inhibitory mechanisms within the ATL, revealed by the decrease in z-scored average ATL gamma activity during action discrimination (Fig. 3b, d, f), and at the end of the response during actor discrimination (Fig. 3a, c, e). Such decreases in gamma power have been recently shown to correspond to inhibition in single neurons^50^.

The correlation of gamma power levels with the RTs revealed an antagonistic relationship between left and right ATL, enriching the repertoire of interhemispheric interactions beyond specialization^16,51^ and cooperation^11^. Indeed the interhemispheric difference in correlation sign was significant, even if the correlation was significant in only a few individual leads. This result suggests that in the actor discrimination task left and right ATL operate together as a push-pull mechanism, an organization well documented at lower levels of the visual system, such as MT^52^ or V1^53^. The benefit of such a mechanism, implying that the left and right ATLs act in opposite fashion upon downstream brain structures generating the motor responses, is an enhancement of the response dynamics, resulting here in shorter RTs. This further underscores the strong links between the ATL activation and behavior, and reconciles the specialization of R and L ATL^16^ with their role as a single bilateral hub^11^.

## Methods

### Patients

Stereo electroencephalography (sEEG) data were collected from 24 patients (13 male, 11 female, age 18–49, mean 31 years, Supplementary Tables 1 & 2) suffering from drug-resistant focal epilepsy. These patients were stereotactically implanted with intracerebral electrodes as part of their presurgical evaluation, at the Claudio Munari Centre of Epilepsy Surgery. The strategy of implantation was based on the presumptive location of the epileptogenic zone (EZ), derived from clinical history, examination of noninvasive long-term video-EEG monitoring, and neuroimaging. Patients were fully informed regarding the electrode implantation and sEEG recordings. The present study received the approval of the Ethics Committee of Niguarda hospital (ID 939-2.12.2013) and informed consent was obtained from all patients in the study. Intracerebral recordings were performed according to sEEG methodology to define the cerebral structures involved in the onset and propagation of seizure activity^54–56^.

### Inclusion criteria

Patient selection was based on a series of stringent anatomical, neurophysiological, neurological, and neuropsychological criteria, with the specific aim of minimizing the recording of any data from pathophysiological and functionally compromised sectors of the brain tissues.

Anatomical criteria: only patients whose magnetic resonance imaging (MRI) showed no anatomical abnormalities, or only very restricted anomalies (e.g., focal cortical dysplasia) were included in the study. The anatomical assessment was carried out in two independent ways: (a) by clinical investigation of the MRI of the patient by experienced neurologists and neurosurgeons, and (b) at the computational level by means of a warping procedure allowing us to identify, per patient, the brain regions not fitting the FS-LR template. The maximal deformation^57^ mapped in temporal lobe across our 24 patients was 8 mm.

Neurophysiological criteria: this examination includes the inspection of the EEG tracks at rest, during wakefulness and sleep from both intracranial and scalp EEG. Pathological activity is characterized by the presence of epileptic discharge at the seizure onset, but epileptic spikes may be present in leads exploring the regions surrounding the EZ during the interictal periods. Since epileptic spikes could affect the quantification of task- and stimulus-related gamma reactivity, each trial presenting an interictal epileptic discharge (IED) at any latency during the stimulus presentation was removed (Supplementary Table 3). Besides inspecting the spontaneous EEG activity, the neurophysiological investigation also included an assessment of the normal reactivity of both intracranial and scalp EEG to a large set of peripheral stimulations (somatosensory, visual, vestibular, and auditory stimulations) to verify normal conduction times and overall sensory reactivity.

Neurological criteria: no seizure must have occurred, no alteration in the sleep/wake cycle must have been observed, and no change in pharmacological treatment must have taken place within the last 24 h before the experimental recording of a patient included in the study. Neurological examination had to be unremarkable, with in particular no motor or visual deficit.

Neuropsychological criteria: a series of neuropsychological tests was administered by experienced neuropsychologists. The tests focused on the evaluation of the patient skills in language (production, comprehension, and reading), verbal memory, visuospatial memory, visual exploration, executive and attentional functions, visual perception, and abstract reasoning. Among them, we considered of particular relevance five items indexing skills which could impact the ability to carry out the required tasks. Their values are reported for 20/24 patients in Supplementary Table 2. For semantic fluency^58^ the overall score is followed by the ranked index; where a value greater or equal to 2 indicates normal function, a value of 1 indicates a subclinical abnormality, and a value of 0 indicates a pathological dysfunction. The second item, naming, was extracted from the Boston Naming Test^59^ and a score below 20 is considered pathological. For visual exploration^60^ a score below 30 is considered pathological. For the fourth item, attentional matrices^58^, the overall score is followed by the ranked index; where a value greater or equal to 2 indicates normal function, a value of 1 indicates a subclinical abnormality, and a value of 0 is considered pathological. Lastly, face recognition was evaluated with the Benton Facial Recognition Test^61,62^ where a value outside the normed range (41–54) is considered pathological.

Results of most of these tests fell within normal ranges for the great majority of the patients. This also held true for those patients from whom doubly-specific ATL leads were obtained (Supplementary Table 3), with the exception of patient 8 who failed on the semantic fluency test, and patient 9 who was borderline on the face recognition test.

### Localization of recording sites with respect to lesions and epileptogenic zone

Only patients presenting with no anatomical alterations (*n* = 18) or with only small abnormalities (*n* = 6), as evident on MRI, were included. Four of the patients with positive MRI showed minimal periventricular nodular heterotopia (three in the temporal lobe, one in the occipital lobe), one patient focal cortical dysplasia (FCD) in the frontal lobe and one hippocampal sclerosis. The epileptogenic zone (EZ) involved parts of the temporal lobe in 17 out of 24 patients (Supplementary Table 3). However, it involved ATL in only 4 patients, for whom only 4 out of 11 doubly-specific leads were located in the EZ (Supplementary Table 3).

### Electrode implantation

Most implantations were unilateral, because clinical evidence generally indicates the hemisphere generating the seizures. Only 3 of the 24 patients were implanted bilaterally, resulting in a total of 27 implanted hemispheres. A number of depth electrodes (range: 12–21; average: 16.5) were implanted in different regions of the hemisphere using stereotactic coordinates. Each cylindrical electrode had a diameter of 0.8 mm and consisted of eight to eighteen 2-mm-long contacts (leads), spaced 1.5 mm apart (DIXI Medical, Besancon, France). Immediately after the implantation, cone-beam computed tomography was obtained with the O-arm scanner (Medtronic) and registered to preimplantation 3D T1-weighted MR images, as described previously^10^. Subsequently, multimodal views were constructed using the 3D Slicer software package^63^, and the exact position in the brain of all leads implanted in a single patient was determined by using multiplanar reconstructions^64^. Leads were identified, following clinical conventions, by a letter corresponding to the electrode shaft, followed by a number starting from the electrode tip.

### Behavioral testing

Setup: Patients were seated 70 cm from a liquid crystal display (Dell P2210, resolution 1680 × 1050 pixels, 60 Hz refresh rate) in a familiar environment. The visual stimuli were generated using a personal computer equipped with an open GL graphics card using the Psychophysics Toolbox extensions^65,66^ for Matlab (The Math Works, Inc.).

Visual stimuli and tasks: The stimuli consisted of 1.166 s video clips showing one of two actors (male or female), standing next to a table and dragging or grasping an object (a blue or red ball) on the table using the right hand. At the start of the video, the hand could be shaped either as a palm or a fist and its position could be either above or on the table. In half of the trials, we increased the size of the video (by 20% of the original) within the aperture. The aperture was created by convolving videos with an elliptic mask causing video clips gradually blur into the black background. Finally, the videos were shown as recorded (actor standing to the right of the table, Fig. 1a), or inverted around the vertical axis (actor on the left side of the table, Fig. 1b). These manipulations resulted in 64 (2^6^) videos which were then presented either in the full length (long trials, Fig. 1c top) or truncated either at the time point corresponding to each individual’s 84% action discrimination threshold (ranging from 200 to 250 ms) or at one of two other points 100 ms earlier or later (short trials, Fig. 1c bottom). The rest of the movie in the short trials was replaced by a dynamic noise which was produced by randomly scrambling every pixel in the display on subsequent frames.

All trials started with a baseline period (1 s), followed by a variable static phase, created by repeating a first video frame, identical for the two actions, for 275, 450, 575, 725, or 875 ms, and then followed by the video displaying either action. If patients could not respond during static and dynamic stimulus presentation, they were given another 2 s to reach a decision before the trial ended. As soon as patients pressed a button during any of the three trial phases (static frame, video, response epoch), the inter-trial period (1 s) started (Fig. 1c).

The trials were organized into four blocks of action (An) or actor (Ar) discrimination tasks. The order of presentation was always An-Ar-An-Ar. Every block consisted of two sub-blocks of 32 trials such that one sub-block contained long trials and the other short ones, presented in pseudorandom order. At the beginning of each sub-block, the instruction saying either ‘action’ or ‘gender’ in Italian was displayed for 5 s. The patients had to follow this instruction in performing either the action or actor two-alternative forced-choice (2AFC) discrimination task by pressing, when ready, either a right or left button with the right hand to indicate their decision. In the first two blocks the original videos were displayed and in the last two, we inverted the videos moving the actor to the opposite visual field. During the trial, a fixation cross was presented near the manipulated object in the center of the screen. During the 1 s inter-trial interval, only the fixation cross was visible.

Patients were instructed to fixate the cross in the center of the screen. In all subjects the experimenter verified that subjects complied with the fixation instruction. Eye movements were recorded in 17 patients using a noninvasive monitor-mounted infrared video system (SMI iView X 2.8.26) sampling the positions of both eyes at 500 Hz. Fixation performance was similar in all patients, with the standard deviation of eye position averaging 0.80° ± 0.10 horizontally and 0.33° ± 0.04 vertically (Supplementary Table 4).

Preliminary procedures: To familiarize patients with action and actor discrimination tasks, we presented them with two familiarization blocks of 30 long trials chosen pseudo-randomly such that they contained equal numbers (15) of the two tested actions and the two tested actors. Patients responded by pressing a button at the end of each trial and received an auditory feedback indicating either a correct (with low pitch tone) or an incorrect (with a high pitch tone) response. The procedure was repeated until patients made fewer than two errors per block. Patients first learned which button corresponded to which actor and then which button corresponded to which action. In addition, every test block was preceded by a short (10 trials) familiarization block reminding patients of the proper button-choices for an upcoming discrimination task.

After completing familiarization blocks, patients viewed a block of 30 pseudo-randomly chosen trials in which videos were truncated at three different time points (150, 250, and 350 ms) from the motion onset with the end of the video replaced by dynamic noise. After collection of the action discrimination performance, the 84%-threshold was estimated for each patient and later used in the experiment to create trials in the short condition.

### SEEG data recording and processing

For each implanted patient, the initial recording procedure included the selection of an intracranial reference, chosen by clinicians using both anatomical and functional criteria. The reference was computed as the average signal of two adjacent leads both exploring white matter. These leads were selected patient-by-patient because they did not present any response to standard clinical stimulations, including somatosensory (median, tibial, and trigeminal nerves), visual (flash), and acoustical (click) stimulations, nor did electrical stimulation evoke any sensory and/or motor behavior, as described previously^10^. The sEEG was recorded with a Neurofax EEG-1100 (Nihon Kohden System) at 1 kHz sampling rate.

The recordings from all leads in the gray matter were filtered (band-pass: 0.08–300 Hz; notch: 50 Hz) to avoid aliasing effects and were decomposed into time–frequency plots using complex Morlet’s wavelet decomposition. Power in the gamma (50–150 Hz) frequency band was extracted within an interval, extending from 1000 ms before the start of the trial to 1000 ms after the latest response by the subject, the timing of which differs between subject and task. This interval was subdivided into non-overlapping 10- or 25-ms bins. Following previous intracranial studies^67,68^, gamma power was estimated for 10 adjacent non-overlapping 10-Hz frequency bands, and averaged. The quality of the data was visually inspected using plots of average gamma power in all trials collected for a given condition, to detect the possible presence of IEDs. All trials/channels in which any IED or other transient electrical artefacts appeared were removed and their average numbers are listed in Supplementary Table 3.

The anatomical reconstruction procedures followed those of Avanzini et al.^10^ and included two basic steps: (1) identifying the recording leads located in the individual cortical surface using the multimodal reconstructions performed in each patient, and (2) importing these locations into a common template, using the warping of the individual cortical anatomy to the fs-LR template.

### Statistical analysis

Behavior data analysis: Accuracy (% correct) and reaction times (RTs) were computed for each of the 24 patients in the four conditions of the design (actor vs action discrimination for long and short trials). Accuracies in the two long conditions and actor discrimination-short were close to perfect performance (97 ± 0.01). In action discrimination-short they were, as expected, close to the individually estimated 84%-threshold (82 ± 0.02). RTs were centered on the mean in all four conditions, except for one patient who had RTs for actor discrimination much longer than those of the other 23 patients (both in long and short trials). This outlier was removed from the analysis of the relationship between ATL latencies and RT (see below). Next, we calculated RT for each of the five static durations and applied two-way analysis of variance (ANOVA) and linear regression analysis with factors task (action, actor) and static duration (275, 425, 575, 725, 875 ms), independently for long and short trials.

sEEG data analysis: The analysis was performed on the average gamma band (50–150 Hz) power sampled with 25 ms bins, and z-scored against the 1 s baseline period, unless specified differently. Each trial was subdivided into three epochs: (1) the 1 s baseline epoch (BEp), ending with static stimulus onset; (2) the static epoch (SEp), defined as the 200 ms time window starting 75 ms after static onset (275 ms was duration of the shortest static phase); (3) the video epoch (VEp), which started at the end of the static presentation (variable across trials) and lasted until a patient gave a response. For each epoch in a trial the mean z-score value was estimated and made available for further analysis. This included analysis of variance (ANOVA), two- and one-tailed *t*-tests, Pearson correlation coefficient and linear regression analysis. When necessary, we corrected for multiple comparison by applying false discovery rate (FDR) correction^69^.

More specifically, we subjected the mean z-scored gamma power in each epoch to a two-way (task × epoch) ANOVA to identify channels in which, for long trials, either the main effect of task or the interaction of task with epoch (or both) were significant, after FDR correction^69^. These channels were considered overall-responsive. Subsequently, we performed post hoc analysis (Tukey’s multiple comparison test) on these overall-responsive leads to identify among them three groups of specific leads: static-, task-, and doubly-specific. These definitions were based on the comparison of mean z-scored gamma power in five epochs in long trials: baseline (BEp) which did not differ between tasks, and SEp and VEp in action and actor tasks. Doubly-specific leads were defined by (1) a mean z-score of SEp in actor task significantly greater than the means of both SEp in action task and baseline, plus (2) a mean z-score of VEp both in action and actor tasks not significantly different from baseline mean. Static-specific leads were defined as leads with (1) an SEp mean z-score greater than baseline mean in both actor and action tasks, while (2) the VEp mean z-score did not differ significantly from baseline mean in either task. Task-specific leads were defined as leads where (1) the SEp mean z-score in actor task was significantly greater than both SEp mean in action task and baseline mean, but (2) the VEp mean z-score in actor task was also significantly greater than VEp mean in action task and baseline mean.

To quantify the magnitude of the differences corresponding to these definitions, we calculated for each lead task (*R*_pa_) and static specificity (*R*_sv_) indices using the long trials. Both indices follow the formula:

1 $$R = \frac{{A_1 - A_2}}{{A_1 + A_2}}$$

where *A*_1_ and *A*_2_ are mean responses to static stimuli (i.e., mean z-score in SEp) for actor/person (p) and action (a) discrimination in *R*_pa_, and are mean responses in static (s) and video (v) epochs (i.e., mean z-score in SEp and VEp) during actor discrimination in *R*_sv_. For both indices *A*_2_ was set to zero where negative. This occurred frequently for the static response during action discrimination in doubly-specific ATL leads (37/46) and task-specific ATL leads (2/2), but not in the other leads (0/15 in static-specific FG leads, and 1/14 in remaining task specific leads). We next identified the position of each lead in a 2D-plane described by *R*_ap_ and *R*_sv_.

As a next step, for each lead selected by the post hoc analysis, we calculated duration and latency of the response to the static stimulus. These were defined by the intersections of the z-scored gamma power time course (10-ms bin resolution) with the 3 SD level above the zero mean, defined on the baseline epoch. Latency was defined as the time interval between the static stimulus onset and the first intersection, and the duration as the time interval between the two intersections. This calculation was performed for the five static stimulus durations independently and also for all durations pooled. We applied two-tailed *t*-tests to compare latencies and durations of leads in different regions across subjects.

For each channel, we next readjusted the time window in SEp to the individual latency and duration values and calculated mean z-score of gamma power for the adjusted window. We correlated this adjusted mean activity with reaction time in actor discrimination across trials.

We also tested across subjects the correlation between the reaction times in the actor discrimination task (long and short trials separately) and latencies. For each subject, we took the average of all leads recorded in that subject (Supplementary Table 3) in either ATL (at least two leads per subject), FG or OTC (one or more leads per subject). To better quantify the relationship between ATL-latency and response time revealed by correlation coefficients, we also performed a linear regression analysis.

### Continuous maps

To provide a continuous view of the topographic pattern of specific leads, we built, following Avanzini et al.^10^, a circular mask (referred to as a disk) using surface nodes (each lead has exactly seven nodes) and geodesic distances^70^ (i.e., the minimum pathway within the gray matter connecting the source and the target nodes). For each cortical node, we defined the nodes within a 1-cm geodesic distance from the original node and weighted the contribution of these nodes by a sigmoid function, defining node weight as a logistic function with unitary amplitude, a steepness of 2 and a midpoint at 7.5 mm. As a result, each node of the cortical mesh was associated with a disk, i.e., a collection of surrounding nodes, in which nodes within 5 mm of the origin were maximally weighted, and those between 5 and 10 mm gradually reduced in weight, avoiding edge effects.

Using this approach we computed two different functional variables. The first is cortical sampling density [i.e., number of explored leads per disk]. In this functional variable, cortical regions with a sampling density below two leads (i.e., 14 nodes) were masked out from subsequent analyses. Relative responsiveness [i.e., the number of nodes (leads) for each response pattern (static, task and doubly-specific, identified by the Tukey post hoc test) as a percent of the number of overall-responsive (tested by ANOVA) nodes (leads) within a disk] is the second functional variable. This variable indexes the degree to which an area responds with a given specificity pattern, based on the time course of lead activations which escapes detection by most of current neuroimaging techniques.

These density and relative responsiveness maps were plotted using CARET software^70^ (www.nitrc.org/projects/caret) and directly compared with retinotopic regions defined in Abdollahi et al.^21^, and with the coordinates of the anterior and posterior FFA^20^, and ATFP^8^ sites.

### Code availability

Custom code used to generate the findings of the study is available upon reasonable request.

## Supplementary information

Supplementary Information

## Data Availability

The data that support the findings of this study are available from the corresponding author upon reasonable request.

## References

[1] Rice A, Phillips PJ, Natu V, An X, O’Toole AJ (2013). Unaware person recognition from the body when face identification fails. Psychol. Sci..

[2] Mouchetant-Rostaing Y, Giard MH, Bentin S, Aguera PE, Pernier J (2000). Neurophysiological correlates of face gender processing in humans. Eur. J. Neurosci..

[3] Kaul C, Rees G, Ishai A (2011). The gender of face stimuli is represented in multiple regions in the human brain. Front. Hum. Neurosci..

[4] Wiese H, Kloth N, Güllmar D, Reichenbach JR, Schweinberger SR (2012). Perceiving age and gender in unfamiliar faces: an fMRI study on face categorization. Brain Cogn..

[5] Visser M, Jefferies E, Embleton KV, Lambon Ralph MA (2012). Both the middle temporal gyrus and the ventral anterior temporal area are crucial for multimodal semantic processing: distortion-corrected fMRI evidence for a double gradient of information convergence in the temporal lobes. J. Cogn. Neurosci..

[6] Tsao DY, Moeller S, Freiwald WA (2008). Comparing face patch systems in macaques and humans. Proc. Natl Acad. Sci. USA.

[7] Rajimehr R, Young JC, Tootell RB (2009). An anterior temporal face patch in human cortex, predicted by macaque maps. Proc. Natl Acad. Sci. USA.

[8] Nasr S, Tootell RBH (2012). Role of fusiform and anterior temporal cortical areas in facial recognition. Neuroimage.

[9] Avidan G (2014). Selective dissociation between core and extended regions of the face processing network in congenital prosopagnosia. Cereb. Cortex.

[10] Avanzini P (2016). Four-dimensional maps of the human somatosensory system. Proc. Natl Acad. Sci. USA.

[11] Lambon Ralph MA, Jefferies E, Patterson K, Rogers TT (2017). The neural and computational bases of semantic cognition. Nat. Rev. Neurosci..

[12] Rice GE, Lambon Ralph MA, Hoffman P (2015). The roles of left versus right anterior temporal lobes in conceptual knowledge: an ALE meta-analysis of 97 functional neuroimaging studies. Cereb. Cortex.

[13] Amalric M, Dehaene S (2017). Cortical circuits for mathematical knowledge: evidence for a major subdivision within the brain’s semantic networks. Philos. Trans. R. Soc. Lond. B. Biol. Sci..

[14] Simmons WK, Reddish M, Bellgowan PSF, Martin A (2010). The selectivity and functional connectivity of the anterior temporal lobes. Cerebr. Cortex.

[15] Wang Y (2017). Dynamic neural architecture for social knowledge retrieval. Proc. Natl Acad. Sci. USA.

[16] Woollams, A. M. & Patterson, K. Cognitive consequences of the left-right asymmetry of atrophy in semantic dementia. *Cortex* **107**, 64–77 (2018). 10.1016/j.cortex.2017.11.014 29289335

[17] Peuskens H (2004). Attention to 3-D shape, 3-D motion, and texture in 3-D structure from motion displays. J. Cogn. Neurosci..

[18] Chiu YC, Esterman M, Han Y, Rosen H, Yantis S (2011). Decoding task-based attentional modulation during face categorization. J. Cogn. Neurosci..

[19] Chang L, Tsao DY (2017). The code for facial identity in the primate brain. Cell.

[20] Weiner KS, Grill-Spector K (2013). Neural representations of faces and limbs neighbor in human high-level visual cortex: evidence for a new organization principle. Psychol. Res..

[21] Abdollahi RO (2014). Correspondences between retinotopic areas and myelin maps in human visual cortex. Neuroimage.

[22] Grill-Spector K, Weiner KS, Kay K, Gomez J (2017). The functional neuroanatomy of human face perception. Ann. Rev. Vis. Sci..

[23] Chen Y (2016). The ‘when’ and ‘where’ of semantic coding in the anterior temporal lobe: temporal representational similarity analysis of electrocorticogram data. Cortex.

[24] Britten KH, Newsome WT, Shadlen MN, Celebrini S, Movshon JA (1996). A relationship between behavioral choice and the visual responses of neurons in macaque MT. Vis. Neurosci..

[25] Tang H (2016). Cascade of neural processing orchestrates cognitive control in human frontal cortex. eLife.

[26] Westheimer G, McKee SP (1975). Visual acuity in the presence of retinal-image motion. J. Opt. Soc. Am..

[27] Kelly DH (1979). Motion and vision. II. Stabilized spatio-temporal threshold surface. J. Opt. Soc. Am..

[28] Whitney D, Cavanagh P (2000). Motion distorts visual space: shifting the perceived position of remote stationary objects. Nat. Neurosci..

[29] Jonas J (2016). A face-selective ventral occipito-temporal map of the human brain with intracerebral potentials. Proc. Natl Acad. Sci. USA.

[30] Ferri S, Kolster H, Jastorff J, Orban GA (2013). The overlap of the EBA and the MT/V5 cluster. Neuroimage.

[31] Orlov T, Makin TR, Zohary E (2010). Topographic representation of the human body in the occipitotemporal cortex. Neuron.

[32] Jastorff J, Orban GA (2009). Human functional magnetic resonance imaging reveals separation and integration of shape and motion cues in biological motion processing. J. Neurosci..

[33] Orban GA, Zhu Q, Vanduffel W (2014). The transition in the ventral stream from feature to real-world entity representations. Front. Psychol..

[34] Kravitz DJ, Saleem KS, Baker CI, Ungerleider LG, Mishkin M (2013). The ventral visual pathway: an expanded neural framework for the processing of object quality. Trends Cogn. Sci..

[35] Chatterjee G, Nakayama K (2012). Normal facial age and gender perception in developmental prosopagnosia. Cogn. Neuropsychol..

[36] Pitcher D, Dilks DD, Saxe RR, Triantafyllou C, Kanwisher N (2011). Differential selectivity for dynamic versus static information in face-selective cortical regions. Neuroimage.

[37] Polosecki P (2013). Faces in motion: selectivity of macaque and human face processing areas for dynamic stimuli. J. Neurosci..

[38] Corbo D, Orban GA (2017). Observing others speak or sing activates Spt and neighboring parietal cortex. J. Cogn. Neurosci..

[39] Wong CL, Harris JA, Gallate JE (2012). Evidence for a social function of the anterior temporal lobes: low-frequency rTMS reduces implicit gender stereotypes. Soc. Neurosci..

[40] Liu H, Agam Y, Madsen JR, Kreiman G (2009). Timing, timing, timing: fast decoding of object information from intracranial field potentials in human visual cortex. Neuron.

[41] Shimotake A (2015). Direct exploration of the role of the ventral anterior temporal lobe in semantic memory: cortical stimulation and local field potential evidence from subdural grid electrodes. Cereb. Cortex.

[42] Zhang X, Li Q, Sun S, Zuo B (2018). The time course from gender categorization to gender-stereotype activation. Soc. Neurosci..

[43] Dupuis-Roy, N., Faghel-Soubeyrand, S. & Gosselin, F. Time course of the use of chromatic and achromatic facial information for sex categorization. *Vision Res*. 10.1016/j.visres.2018.08.004 (2018). 10.1016/j.visres.2018.08.004 30201473

[44] Thorpe S, Fize D, Malot G (1996). Speed of processing in the human visual system. Nature.

[45] Rogers TT, Patterson K (2007). Object categorization: reversals and explanations of the basic-level advantage. J. Exp. Psychol. Gen..

[46] Vogels R, Orban GA (1994). Activity of inferior temporal neurons during orientation discrimination with successively presented gratings. J. Neurophysiol..

[47] Movshon JA, Newsome WT (1996). Visual response properties of striate cortical neurons projecting to area MT in macaque monkeys. J. Neurosci..

[48] Lagae L, Maes H, Raiguel S, Xiao DK, Orban GA (1994). Responses of macaque STS neurons to optic flow components: a comparison of areas MT and MST. J. Neurophysiol..

[49] Rust NC, Mante V, Simoncelli EP, Movshon JA (2006). How MT cells analyze the motion of visual patterns. Nat. Neurosci..

[50] Wittig JH (2018). Attention improves memory by suppressing spiking-neuron activity in the human anterior temporal lobe. Nat. Neurosci..

[51] Luzzi S (2017). Famous faces and voices: differential profiles in early right and left semantic dementia and in Alzheimer’s disease. Neuropsychologia.

[52] Ditterich J, Mazurek ME, Shadlen MN (2003). Microstimulation of visual cortex affects the speed of perceptual decisions. Nat. Neurosci..

[53] Palmer LA, Davis TL (1981). Receptive-field structure in cat striate cortex. J. Neurophysiol..

[54] Munari C (1994). Stereo-electroencephalography methodology: advantages and limits. Acta Neurol. Scand. Suppl..

[55] Cossu M (2005). Stereoelectroencephalography in the presurgical evaluation of focal epilepsy: a retrospective analysis of 215 procedures. Neurosurgery.

[56] Cardinale F (2013). Stereoelectroencephalography: surgical methodology, safety, and stereotactic application accuracy in 500 procedures. Neurosurgery.

[57] Van Essen DC (2005). A population-average, landmark- and surface-based (PALS) atlas of human cerebral cortex. Neuroimage.

[58] Spinnler H, Tognoni G (1987). Standardizzazione e taratura italiana di test neuropsicologici. Ital. J. Neurol. Sci..

[59] Kaplan, E., Goodglass, H. & Weintraub, S. *The Boston Naming Test*. (Lea and Febiger, Philadelphia, 1983).

[60] Biancardi A, Stoppa E (1997). Il test delle Campanelle modificato: una proposta per lo studio dell’attenzione in etá evolutiva. Psichiatr. dell’infanzia e dell’adolescenza.

[61] Benton AL, Van Allen MW (1968). Impairment in facial recognition in patients with cerebral disease. Trans. Am. Neurol. Assoc..

[62] Hamsher KD, Levin HS, Benton AL (1979). Facial recognition in patients with focal brain lesions. Arch. Neurol..

[63] Fedorov A (2012). 3D Slicer as an image computing platform for the quantitative imaging network. Magn. Reson. Imaging.

[64] Dale AM, Fischl B, Sereno MI (1999). Cortical surface-based analysis. I. Segmentation and surface reconstruction. Neuroimage.

[65] Brainard DH (1997). The psychophysics toolbox. Spat. Vis..

[66] Pelli DG (1997). The video toolbox software for visual psychophysics: transforming numbers into movies. Spat. Vis..

[67] Vidal JR (2010). Category-specific visual responses: an intracranial study comparing gamma, beta, alpha, and ERP Response Selectivity. Front. Hum. Neurosci..

[68] Caruana F, Sartori I, Lo Russo G, Avanzini P (2014). Sequencing biological and physical events affects specific frequency bands within the human premotor cortex: an intracerebral EEG study. PLoS. One..

[69] Benjamini Y, Hochberg Y (1995). Controlling the false discovery rate: a practical and powerful approach to multiple testing. J. R. Stat. Soc., Ser. B (Methodol.)..

[70] Van Essen DC (2012). Cortical cartography and Caret software. Neuroimage.

