# Supplementary material for: Rapid and specific processing of person-related information in human anterior temporal lobe
Source: Commun Biol. 2019 Jan 4;2:5. doi: 10.1038/s42003-018-0250-0 (PMC6320334; doi:10.1038/s42003-018-0250-0)
Supplement: Supplementary file 1 — Supplementary Information [file 42003_2018_250_MOESM1_ESM.pdf]

Supplementary information for Rapid and specific processing of person-related information in human anterior temporal lobe. Platonov et al

Includes 10 Supplementary figures, 4 Supplementary tables, and supplementary notes.

Supplementary figures

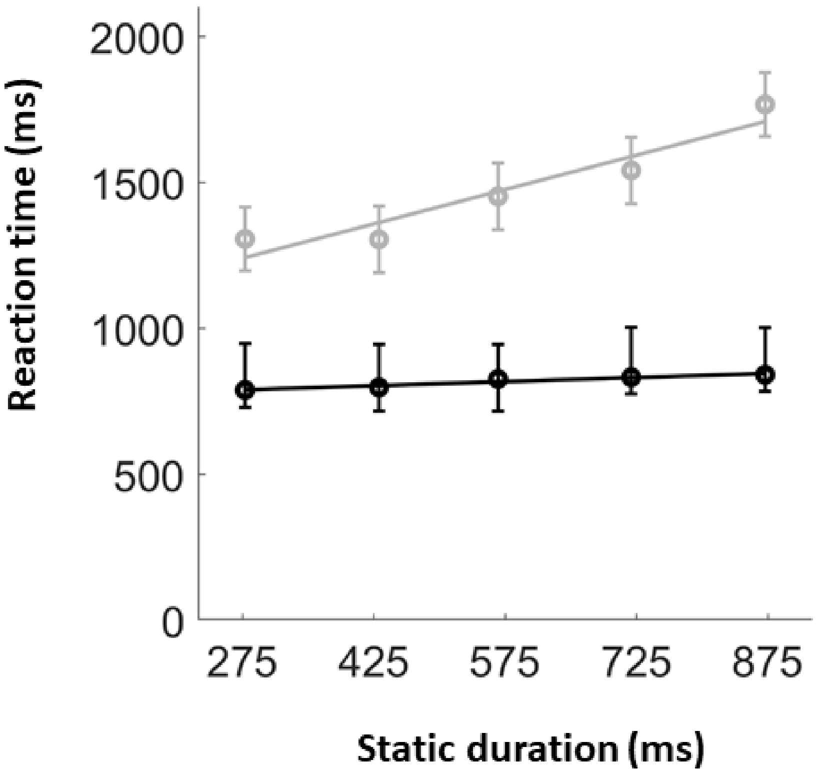

Supplementary figure 1: RT as a function of stimulus duration for short trials. Same conventions as fig 1c. 2-way ANOVA (task x duration): main effect of task $F_{1,2752}$ = 2058, p<0.01, duration $F_{4,2752}$ = 44.7, p<0.01; interaction $F_{4,2752}$ = 29.1, p<0.01.

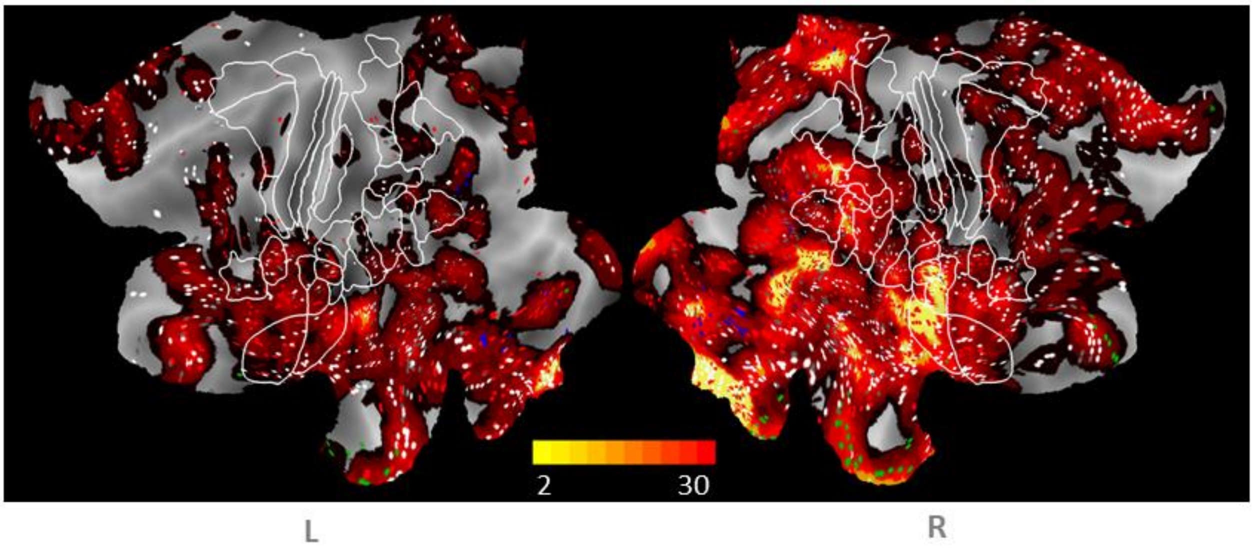

Supplementary figure 2: flatmaps of left (L) and right (R) hemispheres showing coverage. Dots: tested leads. Color code: coverage from 2-30 leads per disc. White outlines: cytoarchitectonic areas in motor, parietal, parietal-opercular and insular cortex[1]

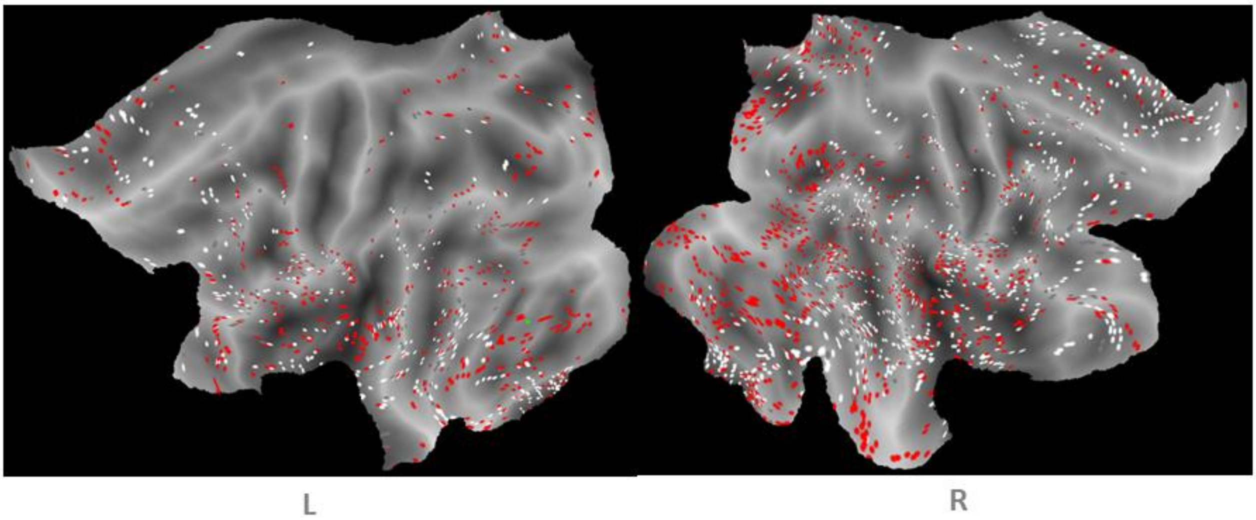

Supplementary figure 3: flatmaps of left and right hemispheres showing overall responsive leads (red dots), unresponsive (white) and not tested (grey) leads.

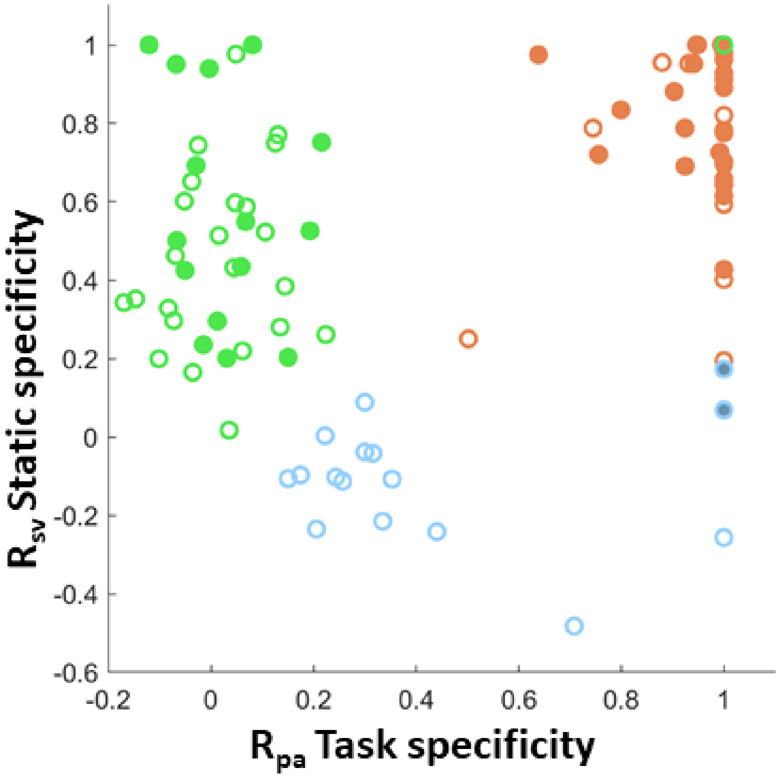

Supplementary figure 4: 2D plot of task and static specificity indices (0= no, 1= complete specificity): orange doubly-specific leads (filled dots: ATL), green: static-specific leads (filled FG), and blue: task-specific leads (filled ATL).

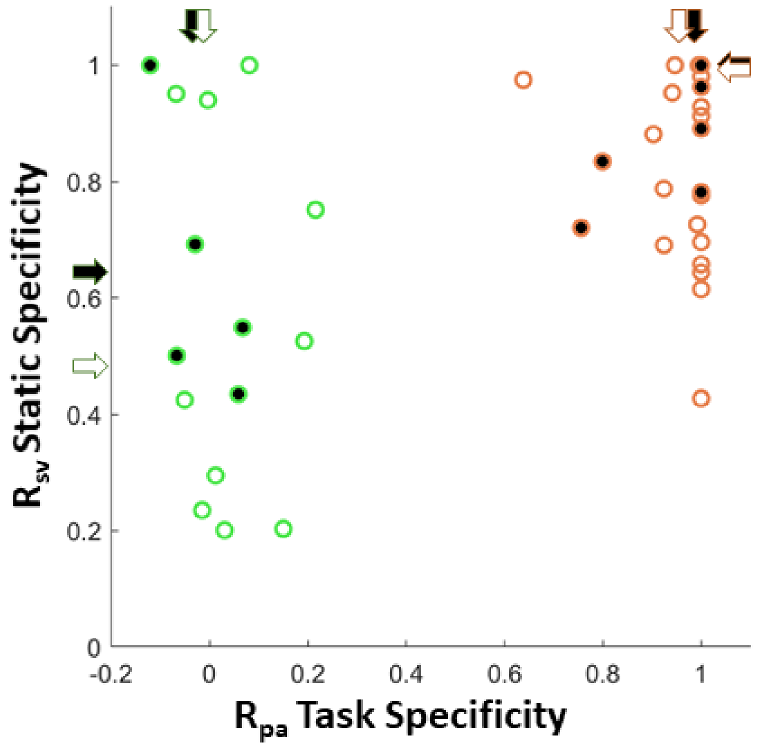

Supplementary figure 5: 2D plot of task and static specificity indices of ATL leads for patients in which ATL was (filled symbols) or was not (open symbols) involved in the epileptogenic zone. Arrows indicate medians.

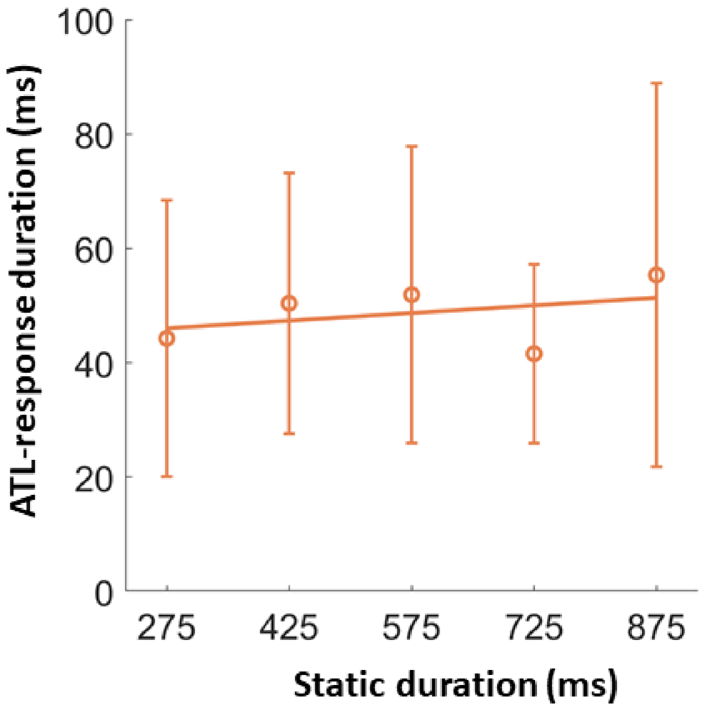

Supplementary figure 6: ATL response duration during actor discrimination as a function of static duration in short trials, error bars= SDs across subjects..

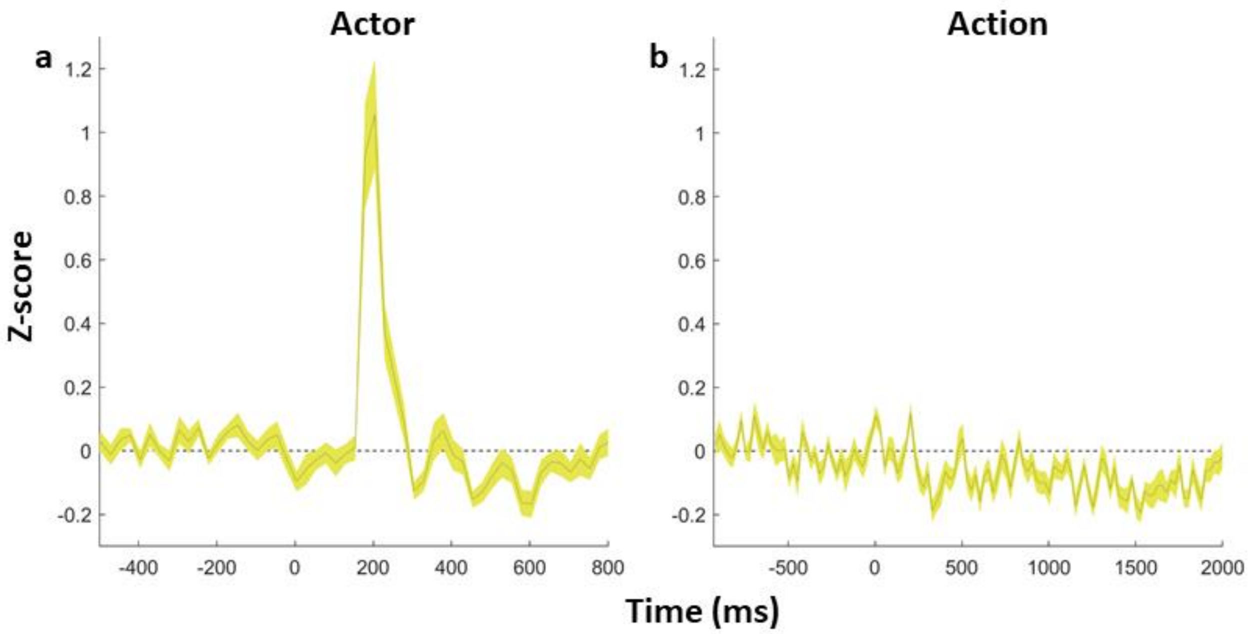

Supplementary figure 7: average time course of OFC leads (n=10) in actor (a) and action (b) discrimination. Hatching: SEs; OFC responses are merely a scaled down version of the ATL responses,

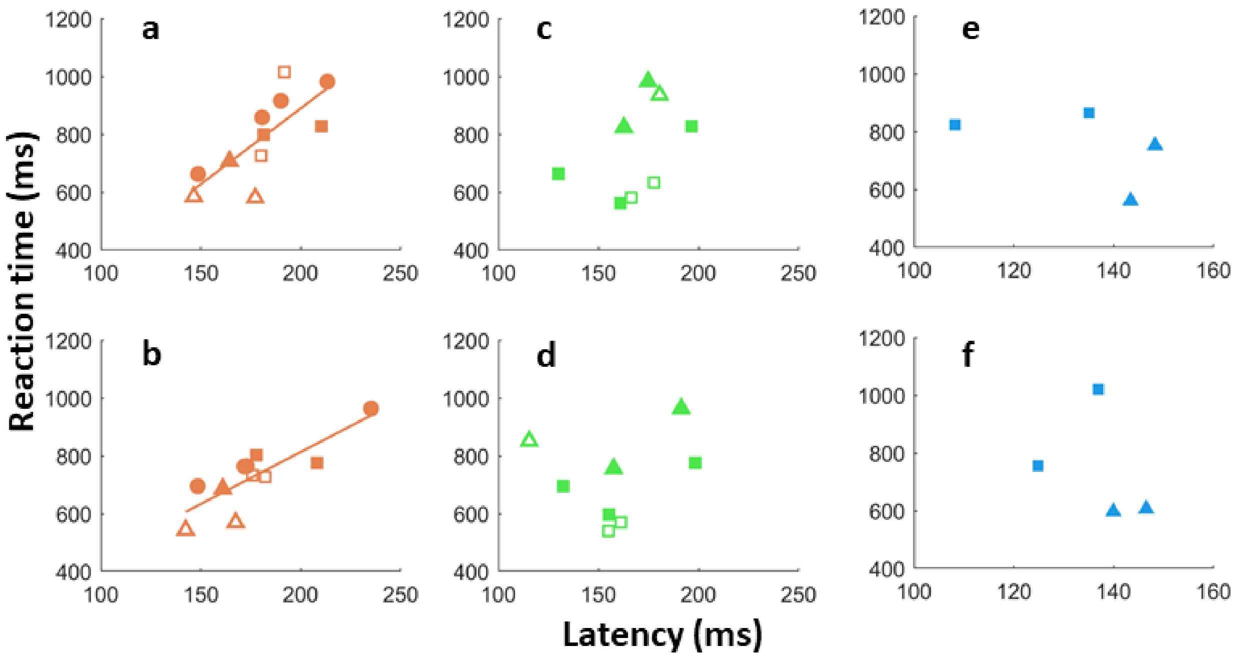

Supplementary figure 8: correlation between RT and latency of specific leads: ATL (a, b), FG (c, d) and OTC (e, f) latencies in both long (a, c, e) and short (b, d, f) trials. Filled and open symbols: right and left hemispheres; symbols indicate number of leads per subject: squares 2 leads in ATL and 1-2 leads in FG and OTC, triangles: 3-4 leads, circles: 5 or more leads.

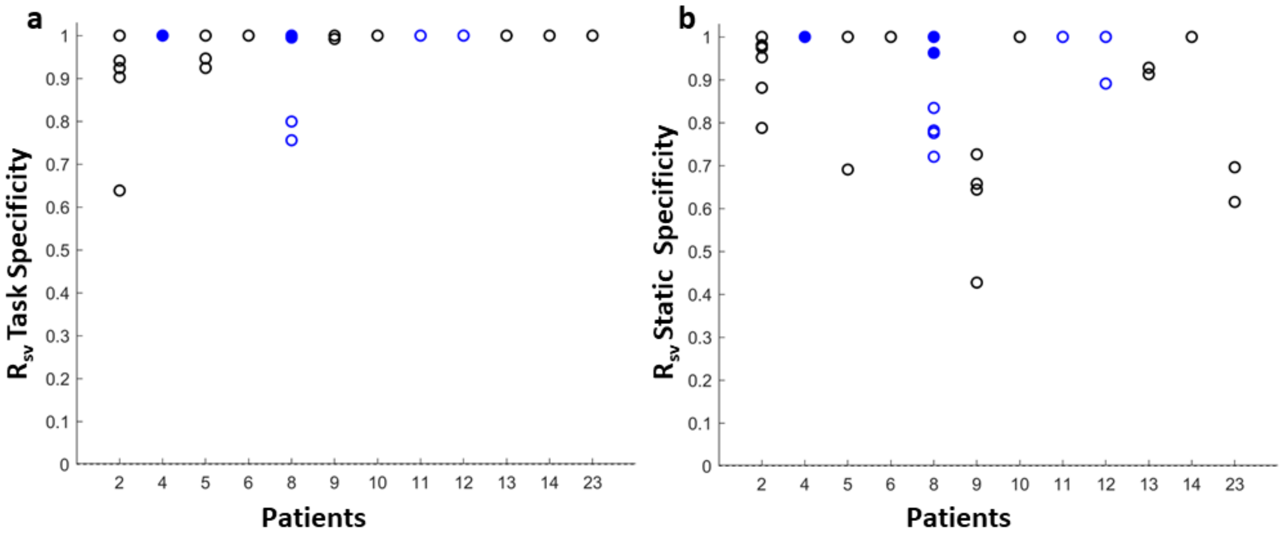

Supplementary figure 9: task (a) and static (b) specific index of ATL leads plotted for the 12 patients in which doubly-specific ATL leads were obtained. Blue: the four patients with EZ in the temporal pole, black: other 8 patients; filled symbols: leads within the EZ, open symbols leads outside the EZ. Patients are indicated by the numbers in supplementary Tables 1-3. Difference between 22 leads in the four patients with EZ in temporal pole and the 24 leads in the remaining patients: static specific index: two-tailed t-test, t_{44} =2.07, p<0.05, ns after correction for four comparisons; task specific index: t_{44} =0.47, p>0.63, ns.

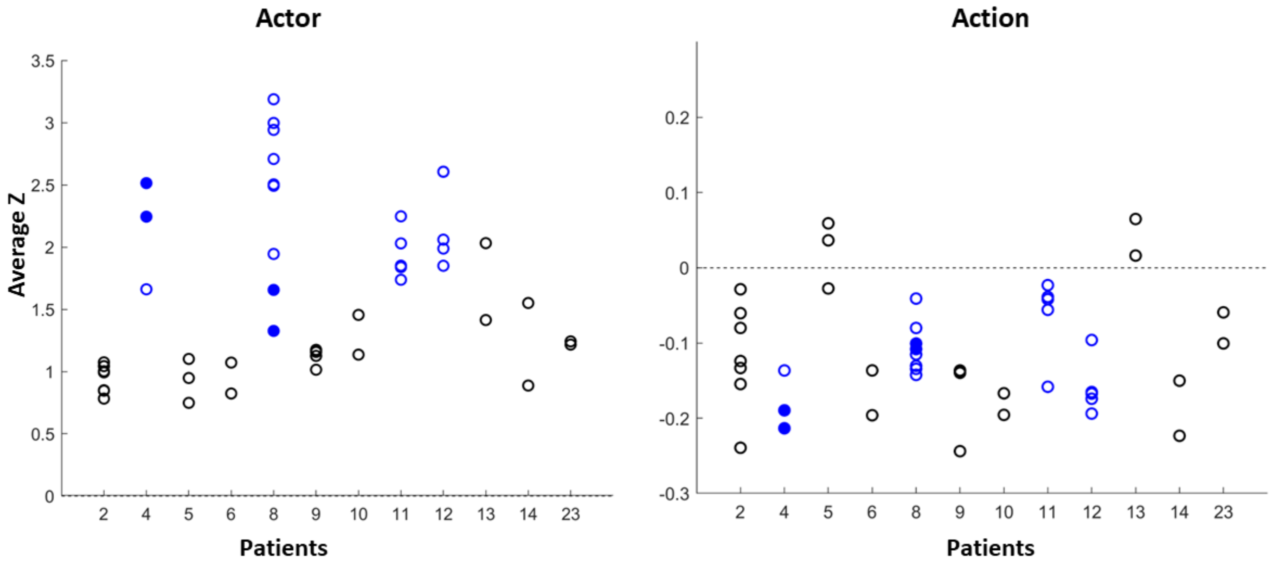

Supplementary figure 10: average z score in the interval 0-800ms after static onset during actor discrimination and in the 500-1500ms interval after static onset in the action discrimination plotted for the different ATL leads of the 12 patients in which doubly-specific ATL leads were obtained. Same conventions as in figure S9. Difference between 22 leads in the four patients with EZ in temporal pole and the 24 leads in the remaining patients: two-tailed t-tests: actor discrimination: t_{44} =9.48, p<0.01; action discrimination: t_{44} =0.55, p>0.58, ns.

Supplementary Tables:

Supplementary Table 1: demographical, clinical, and anatomical variables for all tested patients. In MR column, NEG means negative, PNH indicates peri-nodular heterotopia, FCD indicates focal cortical dysplasia. In Post-surgical pathology, n.a indicates that the patient did not undergo surgical intervention (generally because

Patient ID	Gender	Age	School years	Side	# Elec	MR	Post-surgical Pathology	AEDs
Pt01	M	44,8	13	R	17	NEG	n.a.	CBZ, LTG, PB
Pt02	M	30,1	13	R	18	PNH	n.a.	LEV, CBZ
Pt03	F	23,8	15	R	14	FCD	FCD, type IIA	CBZ, LCS
Pt04	F	43,5	18	L	17	NEG	n.a.	CBZ, ZNS
Pt05	F	25,4	18	L	18	PNH	n.a.	LTG, LEV
Pt06	M	21,6	13	R	17	NEG	n.a.	CBZ, CLB
Pt07	F	29,6	13	L/R	7/12	PNH	n.a.	LEV, CBZ
Pt08	F	23,0	13	R	20	NEG	n.a.	CBZ, FYC
Pt09	F	31,5	13	R	15	NEG	FCD, type IA	LEV
Pt10	M	28,4	13	L	12	HS	n.a.	CBZ, LTG, CLB
Pt11	M	32,8	18	R	13	NEG	negative	LCS, LEV
Pt12	M	34,5	8	R	19	NEG	negative	CBZ, LEV
Pt13	F	30,3	13	L	18	NEG	negative	CBZ, LTG, CLB
Pt14	F	49,3	13	L/R	3/13	PNH	negative	OXC, ZNS, CLOB
Pt15	M	23,6	13	L	12	NEG	negative	ZNS, PB
Pt16	M	33,8	13	R	15	NEG	n.a.	TPM, CLB
Pt17	M	20,9	13	R	15	NEG	negative	CBZ, LEV
Pt18	F	27,0	13	R	19	NEG	negative	LEV
Pt19	M	43,4	8	L	17	NEG	n.a.	LTG, CBZ, CLB
Pt20	F	38,7	8	R	14	NEG	TBP	CBZ, LCS, PB
Pt21	M	19,5	13	R	17	NEG	negative	CBZ, LEV
Pt22	F	26,2	11	L	17	NEG	n.a.	OXC, LEV
Pt23	M	25,4	15	R	16	NEG	TBP	LEV
Pt24	M	42,3	12	L/R	9/12	NEG	n.a.	PHT, LCS

thermocoagulations were successful), FCD indicates that pathology confirmed or indicated a focal cortical dysplasia (Type I is mostly invisible in MR examination, Type II is evident), TBP indicates that surgery is planned. Anti-epileptic drugs (AEDs) include CBZ (Carbamazepine), LTG (Lamotrigine), PB (Phenobarbital), LEV (Levetiracetam), LCS (Lacosamide), CLB (Clobazam), FYC (Perampanel), ZNS (Zonisamide), OXC (Oxcarbazepine), PHT (Phenytoin), TPM (Topiramate).

Supplementary Table 2: neuropsychological variables of all tested patients

Patient ID	Semantic Fluency	Naming	Visual exploration	Executive functions (attentional matrices)	Face Recognition
Pt01	51/4	24	35	58/3	49
Pt02	48/4	24	35	n.a.	49
Pt03	58/4	24	31	45/2	47
Pt04	34/1	22	35	58/3	49
Pt05	n.a.	n.a.	n.a.	n.a.	n.a.
Pt06	n.a.	n.a.	n.a.	n.a.	n.a.
Pt07	42/3	23	34	57/3	50
Pt08	***29/0***	20	35	57/3	52
Pt09	55/4	24	31	55/2	***39***
Pt10	51/4	24	31	57/3	45
Pt11	n.a.	n.a.	n.a.	n.a.	n.a.
Pt12	57/4	24	34	56/3	47
Pt13	51/4	24	***27***	48/2	45
Pt14	34/1	24	34	59/4	54
Pt15	39/3	24	35	57/3	52
Pt16	30/1	22	32	52/2	45
Pt17	***29/0***	23	***22***	52/2	52
Pt18	***28/0***	24	33	60/4	45
Pt19	29/1	24	34	42/1	47
Pt20	38/2	21	35	56/3	41
Pt21	45/4	24	35	58/3	52
Pt22	***17/0***	***19***	***25***	38/1	50
Pt23	n.a.	n.a.	n.a.	n.a.	n.a.
Pt24	53/4	24	30	44/1	***24***

Supplementary Table 3: location of the epileptogenic (EZ) zone, number of doubly-specific ATL leads, their overlap with EZ, number of static specific FG leads, mean and range of trials removed across all leads, and across ATL leads.

Patient ID	Side	EZ	Doubly specific ATL leads	Overlap ATL - EZ	Static specific FG leads	Rejected trials (total)	Rejected trials (ATL)
Pt01	R	Basal temporal	0	0	3	2 (0/24)	n.a.
Pt02	R	Mesial Temporal	7	0	0	1 (0/14)	4 (3/9)
Pt03	R	Fronto-parietal operculum	0	0	0	6 (0/25)	n.a.
Pt04	L	***ATL - Hippocampus***	3	2	0	3 (0/20)	3 (2/3)
Pt05	L	Inferior Temporal	3	0	1	2 (0/22)	7 (7/8)
Pt06	R	Frontal operculum	2	0	0	4(0/32)	4 (2/5)
Pt07	L/R	Lateral Occipital	0	0	0/1	1 (0/4)	n.a.
Pt08	R	***Anterior Insula, Orbito-Frontal, ATL***	9	2	2	13 (1/38)	22 (13/35)
Pt09	R	Lateral Occipito-Temporal	4	0	0	11 (0/35)	10 (8/14)
Pt10	L	Mesial Temporal	2	0	0	20 (3/63)	34 (32/36)
Pt11	R	***ATL***	5	0	0	14 (2/50)	38 (34/42)
Pt12	R	***ATL - Hippocampus***	5	0	3	6 (0/35)	8 (7/9)
Pt13	L	Orbito-Frontal	2	0	0	8 (0/30)	1 (0/2)
Pt14	L/R	Inferior and Middle Temporal Gyri (right)	0/2	0	0/1	2 (0/11)	0 (0/0)
Pt15	L	Superior Temporal Gyrus	0	0	1	9 (1/20)	n.a.
Pt16	R	unknown	0	0	0	19 (1/61)	n.a.
Pt17	R	Lateral Occipito-Temporal	0	0	0	17 (17/18)	n.a.
Pt18	R	Mesial Temporal	0	0	0	10 (1/62)	n.a.
Pt19	L	Superior Temporal Gyrus	0	0	3	19 (1/53)	n.a.
Pt20	R	Lateral Occipito-Temporal	0	0	0	13 (0/47)	n.a.
Pt21	R	Basal Occipito-Temporal	0	0	0	10(8/11)	n.a.
Pt22	L	unknown	0	0	0	10 (1/41)	n.a.
Pt23	R	Frontal	2	0	0	2 (0/14)	4 (3/4)
Pt24	L/R	Bilateral Mesial Temporal	0	0	0	6 (0/45)	n.a.

Supplementary Table 4: standard deviations of the horizontal and vertical eye position in the two tasks (long trials)

P	Action		Person	
	**Horizontal**	**Vertical**	**Horizontal**	**Vertical**
1	0.26	0.46	0.16	0.12
3	1.07	0.47	0.93	1.23
7	0.27	0.26	0.61	0.13
**8**	1.56	0.65	1.19	1.73
**9**	1.08	0.86	1.37	1.32
**10**	1.41	0.63	2.04	0.38
**11**	1.91	0.27	2.01	0.64
**12**	0.36	0.44	0.31	0.19
**13**	0.53	0.54	0.55	0.99
**14**	0.48	0.14	0.53	0.19
16	0.98	0.61	0.89	0.86
17	2.14	0.82	3.25	1.58
18	0.38	0.32	0.64	0.59
19	2.97	0.3	3.44	1.54
20	1.35	0.79	1.2	0.8
22	0.68	0.46	1.01	0.45
24	0.42	0.32	0.48	0.59
	1.05±0.19	0.49±0.05	1.21±0.23	0.78±0.13

SD of horizontal eye position did not differ between the 2 tasks (paired t-test, t_{16} =1.90,p>0.07), but SD of vertical position did (paired t-test, t_{16} =2.62,p<0.02). The numbers in bold indicate patients with ATL leads. Supplementary Figs S9, 10 show that the difference in SD of vertical eye position did not affect the ATL functional properties.

Supplementary Notes.

By concentrating on patients without structural cortical abnormalities in the temporal lobe (Supplementary Table 1, methods), and by removing trials with IEDs, we obtained ATL recordings that can be considered to reflect normal physiological properties of the region, at least for the properties investigated. Indeed, task and static specificity were similar (supplementary figure 9) in the 4 patients with the EZ in the temporal pole, and the 8 remaining patients (Supplementary Table 3). The same held true for the tonic inhibition during action discrimination. In fact task specificity and underlying inhibition was slightly stronger in the four patients with EZ in the temporal pole. Only the response during person discrimination turned out to be significantly (after correction for four comparisons) stronger in those four patients compared the 8 remaining subjects (supplementary figure 10). Still this could represent differences in recording quality or individual differences, as much as a hyper excitability of the EZ. In fact, the 4 leads identified as located within the EZ, did not yield systematically larger responses than the other ATL leads in the same subjects, favoring the former alternatives. This view was supported by the observation of clinicians that the background activity of the ATL leads was well structured, including in patients in which the EZ was located in the temporal pole.

Supplementary Table 3 lists the number (average and range) of rejected trials (out of a total of 128) for all tested leads of patients and then specifically for the doubly-specific ATL leads. Overall the number of leads rejected is small as it averaged 20 trials (15%) or less in all subjects. Not surprisingly the average number was larger for the doubly-specific ATL leads, but it exceeded 20 trials in only 3 patients, with EZ either in the temporal pole or

nearby mesial temporal cortex. However, for the worst ATL lead (in patient 11) still two thirds of the trials were kept. Thus the ATL recordings in all 12 patients were not only valid (ie reflecting normal physiology) but also reliable (based on the majority of trials).

Supplementary references:

1 Avanzini, P et al. Four-dimensional maps of the human somatosensory system. *Proc Natl Acad Sci U S A.* **113,** E1936-43 (2016).
